# Association of Acute Pancreatitis and Myocardial Infarction: Is the Heart Victim or Culprit? – A Case Report and Review of the Literature

**DOI:** 10.7759/cureus.10697

**Published:** 2020-09-28

**Authors:** Fadoua Mouedder, Jamal El Ouazzani, Noha Elouafi, Zakaria Bazid

**Affiliations:** 1 Cardiology, Mohammed I University/Mohammed VI University Hospital/Epidemiological Laboratory of Clinical Research and Public Health, Oujda, MAR

**Keywords:** acute pancreatitis, acute coronary syndrome, stress cardiomyopathy

## Abstract

Acute pancreatitis can be associated with electrical changes mimicking acute coronary syndrome with normal coronary arteries. The association of acute pancreatitis with ST-segment elevation and elevated cardiac enzymes has been reported in few observations. The pathophysiological mechanisms of this association remain poorly understood.

We report the case of a 63-year-old woman presenting with chest pain, changes in the electrocardiogram and elevated cardiac enzymes with normal coronary arteries associated with acute pancreatitis. Stress cardiomyopathy or Takotsubo syndrome associated with acute pancreatitis was the most likely diagnosis in our case. Stress cardiomyopathy should be considered a possibility in case of patients with acute pancreatitis who present with clinical signs suggestive of acute coronary syndrome.

## Introduction

Acute pancreatitis is an acute inflammatory process of the pancreas clinically characterized by epigastralgia, accompanied by elevated pancreatic enzymes [1]. Acute pancreatitis can have several complications including myocardial infarction with normal coronary arteries [2]. The association of acute pancreatitis with ST segment elevation has been reported in few observations and many pathophysiological mechanisms were incriminated such as coronary spasm and hydroelectrolytic disorders [3]. Performing coronary angiography is of great importance to avoid side-effects of antithrombotic therapies in the absence of myocardial infarction.

Herein, we report the case of a patient presenting with chest pain, changes in the electrocardiogram and elevated cardiac enzymes with normal coronary arteries concurrently with acute pancreatitis. Through this observation and based on literature data, we will highlight the possible explanations for this association which may have stress cardiomyopathy as a physiopathological basis.

## Case presentation

A 63-year-old woman with untreated hypertension for 10 years and diabetes on oral antidiabetic drugs, was hospitalized with chest pain radiating to shoulder area which lasted longer than 30 minutes. After a detailed interrogation, she complained of vomiting and epigastric pain for two days before her admission.

**Figure 1:**
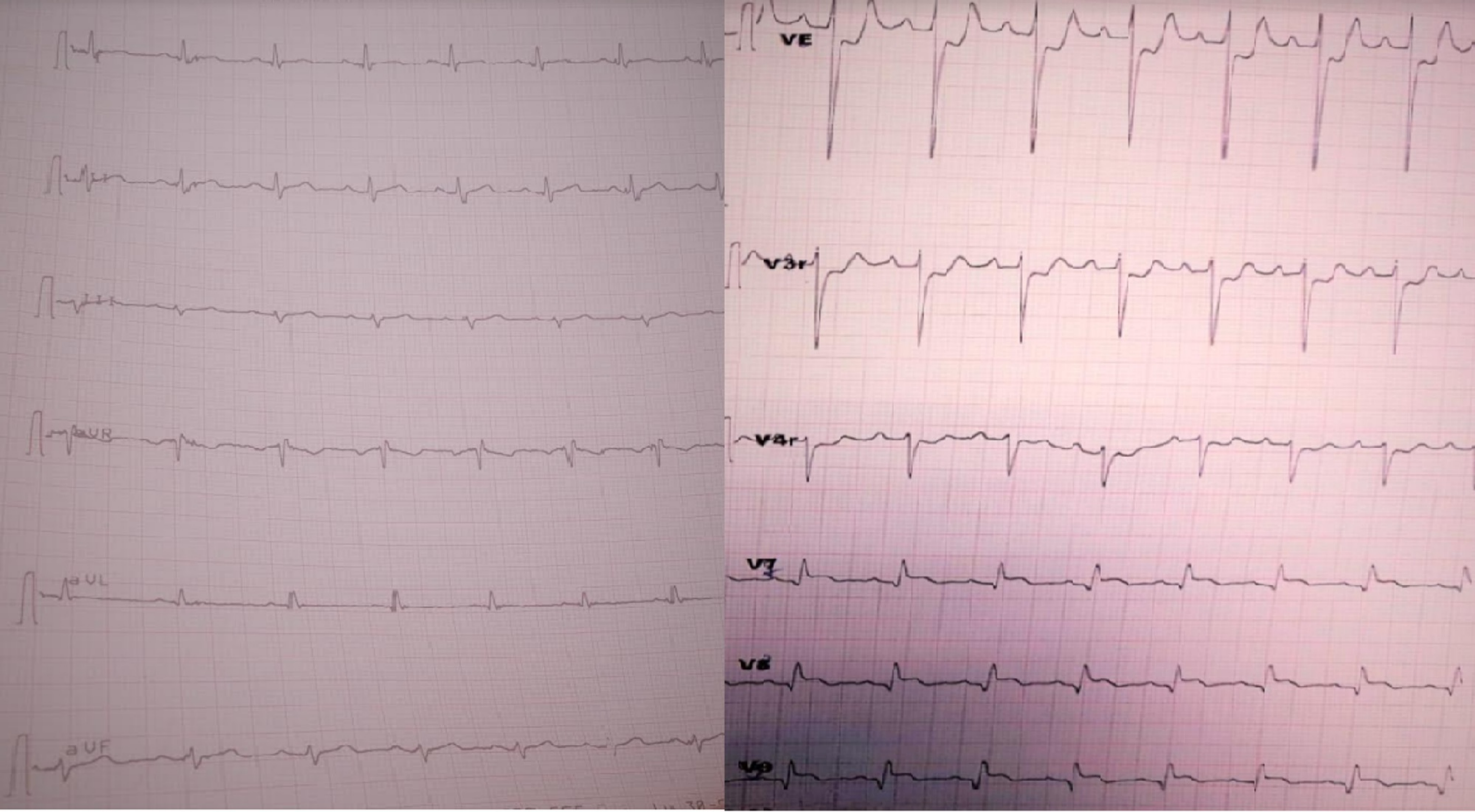
Electrocardiogram performed at admission showing segment ST elevation in posterior leads

On arrival at the emergency department, her pulse rate was 100 beats/min, blood pressure was 120/70 mmHg, and body temperature of 36.2°C. Physical examination revealed tenderness over the epigastric area. Electrocardiogram (ECG) performed on admission showed ST-segment elevation in the posterior leads (V7, V8, V9) without reciprocal changes or pathologic Q waves (Figure 1).

With laboratory analyses, troponin was 83 times higher than the upper limit of normal, thus the diagnosis of acute coronary syndrome with ST segment elevation in the posterior leads was retained and the patient was hospitalized in cardiac care unit for further intensive care. Echocardiography found lateral hypokinesia with left ventricular ejection fraction (LVEF) estimated at 45% without pericardial effusion.

In view of the regression of chest pain, the patient did not benefit from thrombolytic therapy, treatment with antithrombotics was started combining aspirin, clopidogrel and enoxaparin. Given the fact that primary angioplasty was not available at that hour, coronary angiography was performed the following day showing normal coronary arteries. Methergin test was not performed.

**Figure 2:**
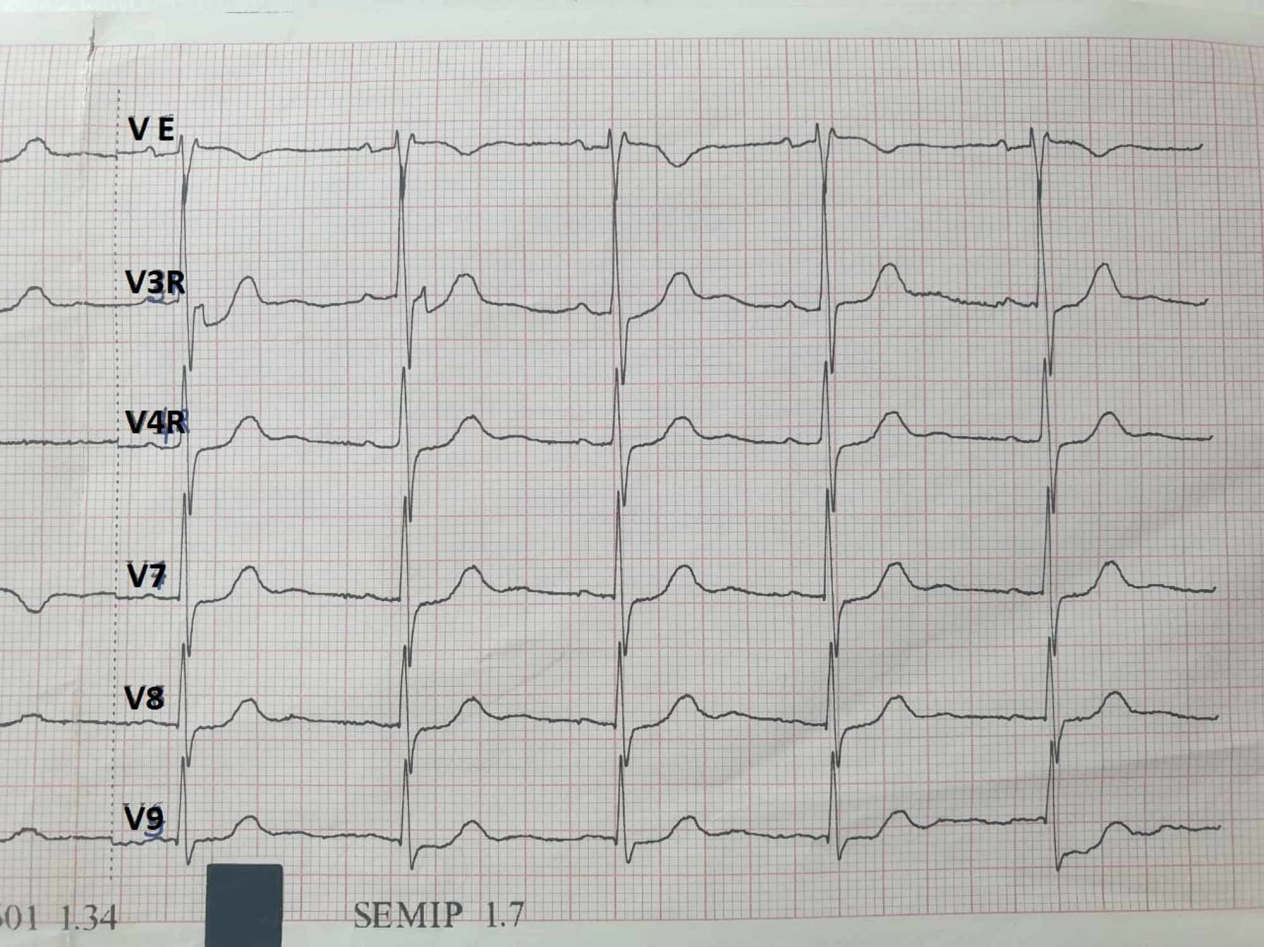
Repeated electrocardiogram (ECG) showing regression of ST segment elevation without pathologic Q waves or negative T waves in posterior leads

Due to increased epigastric pain, an ECG was performed showing regression of ST segment elevation without pathologic Q waves or negative T waves in the posterior leads (Figure 2).

**Figure 3:**
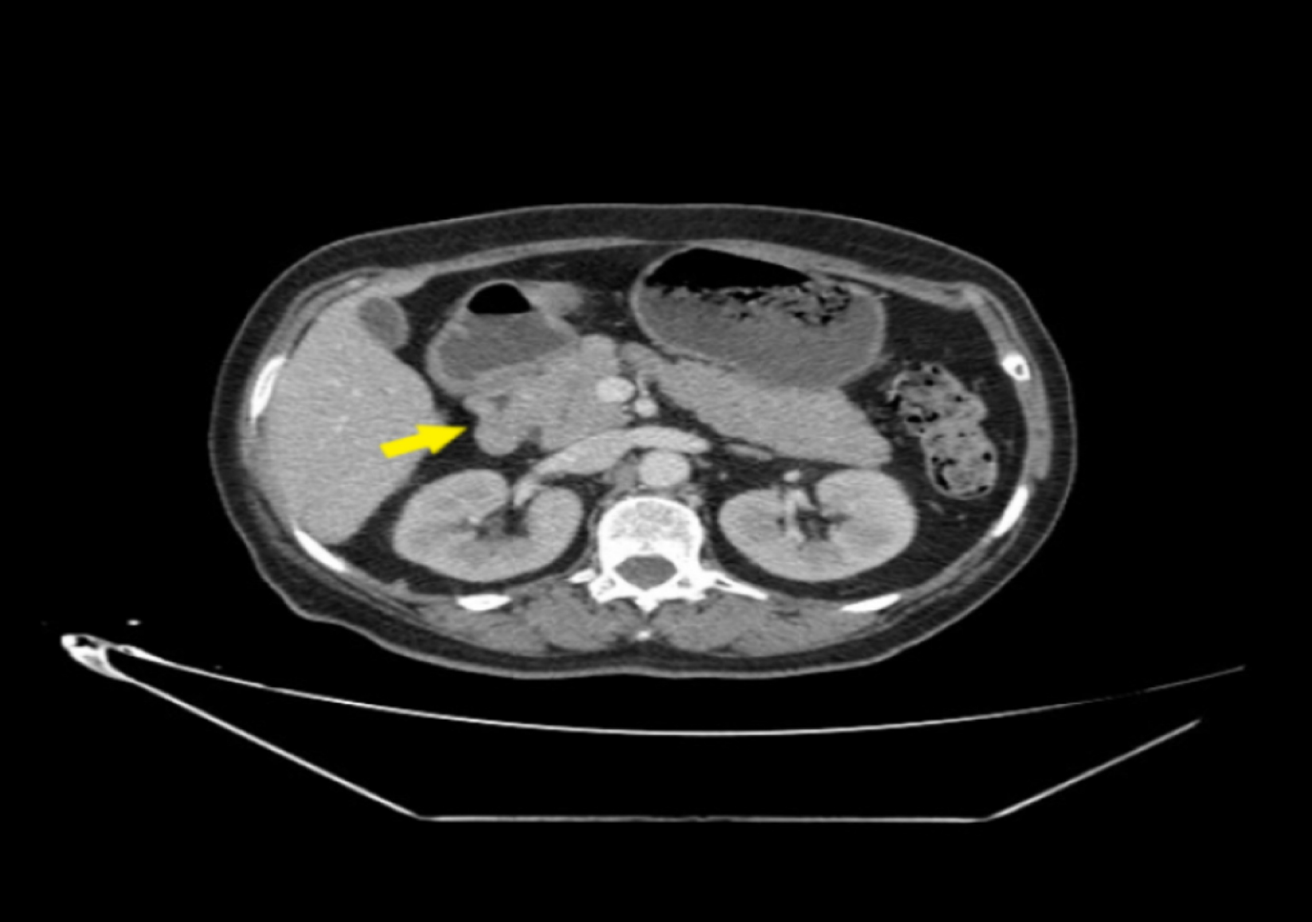
Abdominal computed tomography showing Grade B acute pancreatitis: head of pancreas

**Figure 4:**
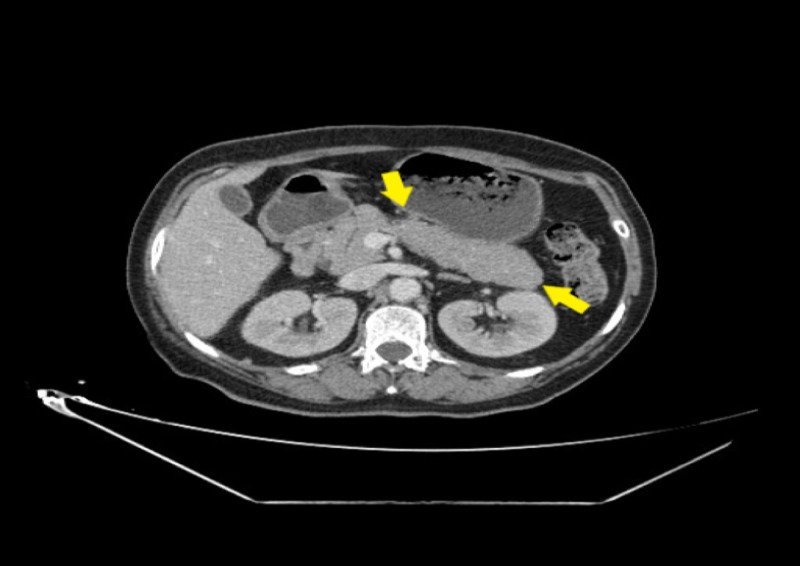
Abdominal computed tomography showing Grade B acute pancreatitis: body and tail of pancreas

Repeated echocardiography showed LV with normal systolic function (LVEF at 60%). Lipase level was seven times higher than the upper limit of normal. Abdominal tomography demonstrated Balthazar grade B pancreatitis, without vesicular lithiasis (Figures 3, 4).

Due to the lack of facilities in our setup to perform cardiac MRI, it was done 10 days after the patient's discharge, objectifying an LV of good global and segmental systolic function with an LVEF calculated at 62%. The sequences performed 10 minutes after gadolinium injection showed absence of late enhancement. Echocardiography performed 15 days later showed normal ventricular function.

## Discussion

Acute pancreatitis can have many complications - local ones such as pancreatic necrosis, abscess or pseudocyst, as well as systemic complications which include pulmonary, renal and cardiovascular abnormalities. Hypovolemia, shock and pericardial effusion are the main cardiovascular events [4]. Pericardial effusion in acute pancreatitis may be secondary to the reaction of pancreatic enzymes transported by the lymphatic vessels on the pericardium or fistulous connection between abdominal and pericardial cavities but also necrosis of vascular walls in fatty areas or necrosis of the subpericardial fat [5].

Acute pancreatitis can also be accompanied by many electrocardiographic changes, for instance arrhythmias, conduction disorders, and changes in the T wave as well as ST-segment elevation simulating myocardial infarction which is rare and occurs especially in the inferior leads [2,6].

The association of acute pancreatitis with ST segment elevation has been rarely described. The precise mechanism remains unknown, it would be probably multifactorial and in most cases cardiac enzymes, echocardiography data and coronary angiographic findings show that it is not myocardial infarction.

It is important to establish a differential diagnosis of real and pseudo myocardial infarction since the therapeutic management will be different in each of these situations. The treatment of myocardial infarction is based on antithrombotics that can lead to disastrous consequences in case of misdiagnosis, especially if it is associated with pancreatitis [7]. Performing coronary angiography is of great importance to avoid side-effects of antithrombotic therapies.

It was not until 1925 that ST segment elevation complicating acute pancreatitis was described for the first time [8]. Many pathophysiological mechanisms were incriminated: coronary spasm, fat embolism, vagal stimulation, hydroelectrolytic disorders and toxic effects of proteolytic enzymes leading to myocardial necrosis [9]. Patel et al. described in 1994 the association of transient regional wall-motion abnormality and normal epicardial coronary arteries with segment ST elevation during acute pancreatitis; they linked it to vagal stimulation [10]. Ro et al. for their part explained it by a coronary spasm [9].

According to several authors enzymes released during pancreatitis may be responsible for local and transient hyperkalemia leading to a depolarization block and thus causing abnormalities of the T wave or ST-segment elevation [11].

Cardiac involvement in acute pancreatitis may be due to stress causing catecholamine discharge, which is part of Takotsubo syndrome or stress cardiomyopathy [12].

Takotsubo syndrome was described in 1990. It is characterized by ventricular dysfunction in absence of abnormality of the coronary arteries. It can have the same clinical presentation as acute coronary syndrome [13]. The current criteria for diagnosing this syndrome according to the European Society of Cardiology (ESC) include: transient LV regional wall motion abnormalities such as hypokinesia, akinesia or dyskinesia, usually an emotional, physical or combined trigger, changes in the ECG (ST segment elevation, T-wave flattening, prolongation of the QTc interval), modest elevation in cardiac troponins with often a significant elevation of brain natriuretic peptide (BNP), absence of a significant coronary artery anomaly and of arguments in favor of myocarditis [14].

The association of stress cardiomyopathy and acute pancreatitis has been rarely described. Nine cases were previously reported, seven out of nine patients were women and age over 55 years [15,16]. They all presented, often with chest pain and dyspnea a week after having a pancreatitis, troponin level was high in all patients. Regarding ECG changes, there was T-wave flattening in four patients, especially in the anterior leads and ST segment elevation was noted in three patients. In most cases echocardiography objectified apical LV akinesis with hyperkinesis of the basal area and they were treated mainly with heart failure drugs with normalization of ventricular function after six weeks [15].

Complete recovery of ventricular function and normalization of segmental kinetic disorders is generally seen within eight weeks [16]. More recent studies using two-dimensional echocardiography suggest that ventricular recovery may not be complete [17].

Although the precise pathophysiological mechanisms of Takotsubo syndrome are not fully understood, several studies have shown the role of sympathetic nervous system which, on the occasion of an emotional, physical or combined trigger, releases an excess of catecholamines that are thought to be the cause of myocardial kinetic disorders [18].

The increase in the level of catecholamines leads to modification of the permeability of the sarcolemma, which in turn to accumulation of calcium in the intracellular environment followed by necrosis of the myocytes. The evolution is generally favorable, since myocardial lesions are reversible due to the short duration of exposure to catecholamines [19].

Based on the latest international consensus of experts proposed by the ESC [20], the diagnosis of atypical Takotsubo syndrome located in the postero-basal areas with rapid recovery complicated by acute pancreatitis or, conversely acute pancreatitis complicated by atypical Takotsubo cardiomyopathy are the most likely diagnoses in our case.

## Conclusions

Acute pancreatitis can be associated with electrical modifications mimicking acute coronary syndrome in the absence of coronary artery abnormalities. Coronary angiography avoids the potentially serious consequences of antithrombotic therapy in the absence of acute coronary syndrome. Stress cardiomyopathy should be considered in case of patients suffering from acute pancreatitis who present with clinical signs suggestive of acute coronary syndrome, especially after the data from the latest international expert consensus of the ESC. The pathophysiological mechanisms of this association remain poorly understood. Several hypotheses have been put forward including the action of pancreatic proteolytic enzymes, but further studies are needed to clearly explain this association.
